# Context-dependent medicinal effects of anabasine and infection-dependent toxicity in bumble bees

**DOI:** 10.1371/journal.pone.0183729

**Published:** 2017-08-23

**Authors:** Evan C. Palmer-Young, Alison Hogeboom, Alexander J. Kaye, Dash Donnelly, Jonathan Andicoechea, Sara June Connon, Ian Weston, Kimberly Skyrm, Rebecca E. Irwin, Lynn S. Adler

**Affiliations:** 1 Organismic & Evolutionary Biology, University of Massachusetts, Amherst, Massachusetts, United States of America; 2 Department of Biology, University of Massachusetts, Amherst, Massachusetts, United States of America; 3 Department of Biology, Dartmouth College, Hanover, New Hampshire, United States of America; 4 Department of Applied Ecology, North Carolina State University, Raleigh, North Carolina, United States of America; USDA Agricultural Research Service, UNITED STATES

## Abstract

**Background:**

Floral phytochemicals are ubiquitous in nature, and can function both as antimicrobials and as insecticides. Although many phytochemicals act as toxins and deterrents to consumers, the same chemicals may counteract disease and be preferred by infected individuals. The roles of nectar and pollen phytochemicals in pollinator ecology and conservation are complex, with evidence for both toxicity and medicinal effects against parasites. However, it remains unclear how consistent the effects of phytochemicals are across different parasite lineages and environmental conditions, and whether pollinators actively self-medicate with these compounds when infected.

**Approach:**

Here, we test effects of the nectar alkaloid anabasine, found in *Nicotiana*, on infection intensity, dietary preference, and survival and performance of bumble bees (*Bombus impatiens)*. We examined variation in the effects of anabasine on infection with different lineages of the intestinal parasite *Crithidia* under pollen-fed and pollen-starved conditions.

**Results:**

We found that anabasine did not reduce infection intensity in individual bees infected with any of four *Crithidia* lineages that were tested in parallel, nor did anabasine reduce infection intensity in microcolonies of queenless workers. In addition, neither anabasine nor its isomer, nicotine, was preferred by infected bees in choice experiments, and infected bees consumed less anabasine than did uninfected bees under no-choice conditions. Furthermore, anabasine exacerbated the negative effects of infection on bee survival and microcolony performance. Anabasine reduced infection in only one experiment, in which bees were deprived of pollen and post-pupal contact with nestmates. In this experiment, anabasine had antiparasitic effects in bees from only two of four colonies, and infected bees exhibited reduced—rather than increased—phytochemical consumption relative to uninfected bees.

**Conclusions:**

Variation in the effect of anabasine on infection suggests potential modulation of tritrophic interactions by both host genotype and environmental variables. Overall, our results demonstrate that *Bombus impatiens* prefer diets without nicotine and anabasine, and suggest that the medicinal effects and toxicity of anabasine may be context dependent. Future research should identify the specific environmental and genotypic factors that determine whether nectar phytochemicals have medicinal or deleterious effects on pollinators.

## Introduction

Flowering plants produce a rich diversity of phytochemicals that have multiple functions, including defense against herbivores and pathogens [1,2]. Chemical defenses are present not only in leaf tissue, but also in nectar, pollen, and other floral structures [3–5]. These floral phytochemicals can protect reproductive tissue from florivores, nectar robbers and thieves, and sexually-transmitted pathogens [6–9].

Antimicrobial phytochemicals can counteract infections not only in plants, but also in plant-eating herbivores and pollinators [10–12]. For example, ingestion of phytochemicals at concentrations present in floral rewards such as nectar—which are typically lower than those in other plant parts [13]—can reduce parasite infection intensity in honey and bumble bees [14–16]. These results suggest that some flowers may have medicinal value, and that plant communities could influence infection patterns among pollinators. However, other studies found no medicinal effects of some compounds that previously reduced infection, including nicotine, thymol, and anabasine [17,18]. This variability suggests the existence of factors that alter the potential medicinal effects of phytochemicals on bees. This study considers several candidate factors, including parasite lineage, bee genotype, phytochemical preference, and bee rearing conditions and nutrition.

Parasite lineage or genotype can have strong effects on an infection’s sensitivity to phytochemicals and pharmaceutical drugs [19–21]. One possible difference between the studies that showed negative versus positive results relates to the parasite lineage. In each case, *Crithidia* spp. parasites were collected locally at the time and place of the trial. Parasites, including those of bees such as the trypanosome gut parasite *Crithidia* spp., can exhibit high levels of genetic diversity and recombination [22,23]. However, no study has explicitly assessed variation among pollinator parasite lineages in susceptibility to phytochemicals *in vivo*. Prior studies that showed medicinal [16] vs. non-medicinal or infection-aggravating [17,18] effects of nectar phytochemicals on bee parasites were conducted using different, locally collected, wild lineages of *Crithidia*. An experiment that explicitly tests multiple parasite lineages in parallel will clarify the degree to which parasite lineage determines the antiparasitic activity of phytochemicals in hosts, and how generally useful phytochemicals are likely to be against parasites in genetically diverse pollinator-parasite communities.

Another determinant of the medicinal value of phytochemicals is whether these compounds are actively sought and consumed by infected hosts. Although high phytochemical concentrations often deter bees [24,25], lower phytochemical concentrations can be attractive [26,27]. Phytochemical preferences can also change across ecological contexts. Both herbivores [10,11,28] and bees [29,30] express infection-dependent preferences for anti-parasitic substances. This behavioral plasticity, in which medicinal chemicals are repellent to healthy animals but preferred by infected animals, may enable hosts to minimize the costs of potentially toxic effects of phytochemical consumption while still obtaining medicinal benefits. For a chemical to be considered medicinal, its use should benefit infected individuals [12], although it may harm uninfected individuals. For example, pyrrolizidine alkaloids increased mortality of unparasitized *Grammia incorrupta* caterpillars, but reduced mortality of parasitized caterpillars. In bumble bees *(Bombus impatiens)*, phytochemical consumption can be costly for uninfected bees [31–33]. However, phytochemicals can also ameliorate bee infection [16,34]. Because infection can reduce bee fitness [35,36], consumption of antiparasitic compounds may be selectively beneficial for infected bees. On the other hand, two previous studies that showed antiparasitic effects of phytochemicals did not find that phytochemicals improved survival of infected bees [15,16]; that is, phytochemicals may reduce parasitism, but it remains unknown whether such compounds are beneficial for infected hosts. Ultimately, effects on host fitness, and not on parasites *per se*, determine whether phytochemical ingestion is adaptive [12].

The effects of dietary chemicals on both hosts and parasites may also be modulated by nutritional environment. For example, dietary protein is necessary for host expression of immune genes [37], detoxification pathways that protect hosts from phytochemicals [38], and survival and reproduction [39,40]. However, a well-nourished host may also provide a more suitable environment for parasites [41], which would explain the higher infection levels found in bees fed ample pollen [40,42]. Because of dietary protein’s importance in induction of detoxification genes [38], phytochemical tolerance may be higher in well-nourished hosts, such that phytochemicals are more beneficial in nutritionally rich environments. The effects of nutrition on phytochemical-mediated benefits against infection are important for bees, which have phytochemical-rich diets but may also face periodic food shortages [43,44]. However, no experiments have considered how the effects of phytochemicals on bees and their parasites vary across nutritional contexts.

Four experiments were conducted with *Bombus impatiens* and *Crithidia* to address the context-dependent medicinal value of the nectar alkaloid anabasine and its isomer nicotine for bumble bees. We addressed the following hypotheses:

1. Due to differences in sensitivity among parasite lineages, anabasine reduces infection with some lineages of *Crithidia* but not others. In the Parasite Variation Experiment, we tested for variation in anabasine’s effects across four *Crithidia* lineages.
2. Bees self-medicate, such that relative preference for phytochemicals is increased under conditions of infection. In the Host Preference Experiment, we examined whether infection altered preference for 30% sucrose solution containing either anabasine or nicotine compared to phytochemical-free control solutions.
3. Anabasine consumption is deleterious for uninfected bees, but beneficial for infected bees. In a Life History Experiment, we tested whether anabasine affected infection intensity in microcolonies; and examined whether infection, anabasine, or their interaction affected food consumption, mortality, and microcolony performance.
4. Anabasine consumption has greater benefits in pollen-fed than in pollen-deprived bees. In the Pollen Deprivation Experiment, we tested whether anabasine reduced infection in bees fed sucrose solution but deprived of pollen, and whether consumption or mortality was influenced by infection, anabasine, or their interaction.

### Study system

#### Parasite: *Crithidia*

*Crithidia* is a hindgut trypanosome parasite of bumble bees [45] that is widespread and abundant. Although one study found that infection prevalence may be only 5–10% in newly-emerged queens, infection rates of mature colonies can range from 30% in Switzerland [23] to over 50% in other areas [35,46,47]. Infection can be costly for workers and colonies. *Crithidia* infection hastens mortality in starved workers [36], decreases queen colony-founding success and colony production [35,36], evokes a potentially costly immune response [48], and may alter worker foraging abilities [49] in *Bombus terrestris* and *B*. *impatiens*.

The spread of *Crithidia* infection is aided by transmission at flowers [50,51]. However, flowers may also expose parasites to floral phytochemicals, both directly on floral surfaces, and indirectly in the digestive tract when pollinators consume pollen and nectar [52], both of which are rich in phytochemicals [3–5].

*Crithidia* populations are diverse, and different strains can sexually recombine [22]. Within a given sampling region, the same genotype may seldom be found more than once [23], and three-fourths of infected bees may harbor multiple *Crithidia* genotypes [23]. Different *Crithidia* strains can have markedly different growth rates and levels of infectivity that vary according to host-parasite genotype-genotype interactions [53–55]. *Bombus-Crithidia* host-parasite interactions can be further modulated by host nutrition, such that each strain performs best in a particular host genotype fed a particular dietary sugar concentration [56].

#### Host: Bombus impatiens

We examined *Crithidia* infections in the Common Eastern Bumble Bee, *Bombus impatiens*. Like other bumble bee species in temperate regions, *B*. *impatiens* has an annual colony cycle in which inseminated queens emerge in spring to found new colonies. Colonies increase in size during the growing season as the worker populations increase. In autumn, the colonies switch from the production of workers to the production of reproductive drones and queens [57], and the colony senesces as floral resources diminish. Although *B*. *impatiens* is not in decline, it is a widespread and commercially available model in which to study the dynamics of infection, and may yield insights into the conservation of bumble bee species that are threatened by infection-related decline [44,58].

#### Phytochemicals: Anabasine and nicotine

Anabasine and nicotine are alkaloids found in plants of the genus *Nicotiana*, including in nectar [13,59]. Both compounds are acetylcholine receptor agonists that have strong effects on the insect nervous system, resulting in convulsions and death at high concentrations [60], and have been historically used as insecticides [61–64]. However, bees appear resistant to naturally occurring concentrations of these compounds. Adult honey bee survival was unaffected by concentrations of up to 50 ppm nicotine [65], and colony performance was robust to 6 ppm [32]. Both of these concentrations exceed those typically found in nectar [13,66]. Moreover, several studies have found that nicotine and anabasine reduced *Crithidia* infection intensity in *Bombus impatiens* and *B*. *terrestris* at levels similar to those in floral nectar [15,16], although other studies reported no or variable benefits of nectar alkaloid ingestion [17,18]. Together, these studies suggest that nectar nicotine and anabasine consumption could be protective against parasites but have minimal costs for bees [32,65].

In *Nicotiana* nectar, anabasine can range in concentration from 0–5 ppm in and nicotine from 0–17 ppm [13,59,66]. In this study, for anabasine we used 5 ppm to correspond to the mean anabasine concentration of *N*. *glauca* nectar [66]. For nicotine (used only in the preference trials), we used 2 ppm; nectar concentrations can exceed 8 ppm in *Nicotiana attenuata* [59], but were below 2 ppm in all but one of 32 *Nicotiana* species surveyed [13].

## Materials and methods

### Parasite collection

Our experiments used six different *Crithidia* parasite lineages (defined as groups of parasites collected at the same time and location, and incubated in the same series of laboratory colonies) collected from Massachusetts and Vermont, USA. *Crithidia* lineages were established from wild *Bombus impatiens* collected at four sites near Amherst, Massachusetts and Norwich, Vermont, USA, in September 2014. The Parasite Variation Experiment used four lineages named for their sites of collection: (1) “**HF**” (Hampshire Farm Amherst, MA (42.329918, -72.524552)); (2) “**SG**” (Simple Gifts Farm Amherst, MA (42.407050, -72.529282)); (3) “**SS**” (Stone Soup Farm, Amherst, MA (42.363436, -72.567973); and (4) “**VT**” (US Route 5, Norwich, VT (43.737069, -72.263626)). The Life History Experiment used a parasite lineage that originated from bees caught at the University of Massachusetts Amherst campus (GPS coord: 42.389, -72.522) in September 2013. The Host Preference Experiment used a separate *Crithidia* lineage collected from three sites near Norwich, Vermont, USA in 2013 (GPS coordinates: “Connor”: 44.262, -72.507; “Morse”: 44.283, -72.543; “Sharon”: 43.680, -72.392). The Pollen Deprivation Experiment was conducted with the same lineage used in the Life History experiment [17,18].

The lineages were maintained by serial propagation in deliberately infected laboratory “source” colonies, which were used as sources of infection to inoculate experimental bees from separate, “experimental” colonies. To initiate infection, homogenized intestinal tracts from dissected bees were diluted in water and examined under 400x magnification. Diluted guts of 2–3 infected bees per site were mixed with 50% sucrose and individually fed to workers of the source colonies. When colonies began to produce reproductives (usually after 2–3 months), the infection of each lineage was transferred to young colonies by inoculation of workers and honeypots with diluted gut extracts from infected bees of the previous source colony.

### Colony rearing conditions

We used commercial colonies of *B*. *impatiens* (Biobest, Leamington, ON, Canada), with an initial size of approximately 30–40 workers and one queen. Upon arrival, at least 10 bees per colony were dissected to verify absence of *Crithidia*. Colonies were fed from a reservoir containing 30% w/w sucrose (cane sugar), and given a ~20 g piece of ground multi-floral pollen mixed with 30% sucrose (Koppert Biological Supply, Koppert, MI, USA) every 2–3 days (~1 mL sucrose solution per 3 g pollen). The colonies were kept inside acrylic plastic containers within cardboard boxes (30 cm length x 23 cm width x 25 cm height). The deliberately infected “source” colonies were kept separate from the uninfected “experimental” colonies, from which bees were taken for experiments.

### Parasite variation in resistance to anabasine

To test the effects of anabasine, *Crithidia* parasite lineage, and their interaction on *Crithidia* infection intensity, we used parasites collected from four field sites near Amherst, Massachusetts and Norwich, Vermont, USA in fall 2014. Individual worker bees from commercial colonies were experimentally inoculated with parasites from one of the four lineages and fed 30% sucrose solutions with 5 ppm anabasine (Anabasine treatment) or 30% sucrose alone (Control treatment) for 7 d, at which time bees were dissected to assess infection intensity [17].

#### Experimental inoculations

Adult worker bees newly emerged from pupation, identified by their pale coloration, were removed from experimental colonies daily. Generally, 3–4 experimental colonies were active at any given time, for as long as they continued to produce workers (4–6 weeks). We used 12 colonies over the course of the experiment (n = 10 to 117 bees per colony), which lasted 5 months. Bees were weighed to the nearest 1 mg and moved to 18.5 mL snap-cap clear plastic vials, in which they were kept throughout the experiment (Figure A in S1 File). Vials were incubated horizontally in constant darkness inside a 28°C incubator. To supply bees with sugar water, the open end of a 2 mL microcentrifuge tube was filled with 30% sucrose (500 μL day^-1^), plugged with a ~1 cm long dental cotton wick, and inserted into a hole in the vial’s cap. Bees were transferred daily to clean vials with fresh sucrose solution and a small piece of pollen (~50 mg). Each bee was first incubated for 1–2 d under control conditions. On the third day, bees were starved for 3–4 h, inoculated, and began receiving anabasine treatments.

Within each colony, bees were systematically assigned to anabasine treatments and *Crithidia* lineages as follows: a number was assigned to each bee based on temporal order of eclosion from the pupal clump. Odd-numbered bees were given control solutions; even-numbered bees were given anabasine. On each inoculation date, we prepared a single *Crithidia* inoculum from one of the four parasite lineages, which was used to infect each bee that had emerged 2 d prior. Each lineage was used in one of every four successive days of inoculation.

To prepare the inoculum, intestinal tracts from bees in the “source” colonies were homogenized for 3–4 seconds with a plastic pestle in a 1.7 mL microcentrifuge tube along with 300 μL distilled water. The tube was briefly vortexed, then allowed to rest at room temperature for ~5 h to allow gut contents to settle and parasite cells to swim from the intestine into the supernatant. A 10 μL aliquot of each gut was screened for infection intensity by surveying a 0.02 μL volume in a Neubauer hemocytometer at 400x magnification. Gut extracts of 2–3 bees were diluted to 1200 cells μL^-1^, then mixed with an equal volume of 50% sucrose to make inoculum with 6000 parasite cells per 10 μL droplet. This dose reflects a typical parasite concentration in feces of an infected bee [67]. Experimental bees were fed a 10 μL droplet of inoculum from a micropipette tip.

#### Anabasine treatments and *Crithidia* quantification

Anabasine treatments commenced immediately post-inoculation and were provided throughout the 7 d experiment. Sucrose solutions with anabasine (5 ppm) were made by dissolving the liquid compound (+/- anabasine, 97% purity, Sigma-Aldrich, St. Louis, MO, USA; mixed enantiomers were also used in previous studies of *Crithidia* [16,18]) in 30% w/w sucrose. Solutions were stored at 4°C and remade every 2 weeks. (Stock solutions of nicotine and related alkaloids are stable for >3 months at 4°C [68].) Bees were supplied with 500 μL treatment solution and fresh pollen (~50 mg) daily.

Bees were dissected 7 d post-infection to assess infection intensity using the methods described above (see Experimental Inoculations). *Crithidia* infection normally reaches a maximum by this time [67].

#### Statistical analysis

Analyses were conducted in R version 3.2 for Windows [69]. Linear models were fit by restricted maximum likelihood in R package “lme4” [70]; Cox proportional hazards models were fit with the package “coxme” [71]. Least-squares means and standard error were calculated with package "lsmeans" [72]. Graphs were generated and assembled through R packages “ggplot2”, “cowplot”, and “survminer” [73–75].

For analysis of infection intensity, *Crithidia* counts were ln(x+1)-transformed to improve homogeneity of variance and normality of residuals. A linear mixed-effects model was fitted with *Crithidia* parasite count as response variable; anabasine treatment, *Crithidia* lineage, and their interaction as fixed predictors, bee mass at emergence as a covariate, and experimental colony and inoculation date as random effects. Initially colony was included as a fixed factor to test for colony by anabasine interactions; however, this interaction was non-significant (*p* = 0.40), so the model was reconstructed with experimental colony as a random effect. Bees from two colonies were excluded from the analysis due to insufficient replication (<3 bees per lineage and treatment). The final analysis included 466 bees (n = 49–70 per lineage and diet treatment (Table A in S1 File)).

Effects of diet treatments on survival (46 total deaths among 602 bees, including the two colonies excluded from analysis of infection) were analyzed with a Cox proportional hazards model [71], with death hazard rate as the response variable, anabasine treatment as a fixed predictor, bee mass at emergence as a covariate, and experimental colony as a random effect. Effects of *Crithidia* lineage and anabasine by colony interactions were not included because we had insufficient deaths in each lineage and anabasine treatment to test for an interaction [76]. Models were simplified by chi-squared tests [77] to remove non-significant covariates (*p>*0.15).

### Effects of infection on host preference for phytochemicals

This experiment assessed how infection altered preference for nicotine or anabasine compared to phytochemical-free control solutions. Newly emerged bees were removed and infected 2 d post-emergence with either 6000 *Crithidia* parasite cells in 25% sucrose, or a control solution of 25% sucrose without parasites. Thereafter, bees were maintained in individual vials for 7 d (~27°C, constant darkness), to allow the infection to develop before assessment of preference. During the 7 d incubation period, bees were moved daily to clean plastic vials with fresh 30% sucrose solution (500 μL) and approximately 50 mg pollen moistened with sugar water (1 mL 30% sucrose per 3 g pollen).

Preference experiments were conducted in rectangular sandwich-style deli containers (14 cm x 10.7 cm x 5.1 cm). Each arena was fitted with two 2 mL microcentrifuge tubes, inserted into ports drilled on opposite sides of the arena (Figure B in S1 File). One tube contained a phytochemical-free 30% sucrose solution control solution, and the other contained 30% sucrose solution with added nicotine or anabasine. Although our other experiments focused on anabasine, for this question we also included nicotine, which is also often present in *Nicotiana* nectar [13]. On the side of each tube, we drilled two 2.66 mm diameter drinking holes. At the start of trials, each tube was filled with 1200 μL of the appropriate solution. The tubes were weighed to the nearest 0.0001 g immediately prior to adding bees to the arenas, and again after a 24 h feeding period (constant darkness, 27°C). Consumption was calculated as the difference in weights before and after 24 h feeding. To correct for mass loss due to evaporation rather than consumption, we also recorded changes in mass of tubes in 15 bee-free control arenas, which were incubated in parallel to the preference trials. Mass loss from tubes of the corresponding treatment was subtracted from gross consumption to estimate net consumption. After each trial, bees were dissected to confirm presence or absence of *Crithidia* infection. No infection was found among bees in the uninfected treatment. Bees that had been experimentally inoculated but did not have microscopically detectable *Crithidia* infection were excluded from analysis. The right forewing of all experimental bees was collected for measurement of the marginal cell length, a correlate of bee body size [78] that was used as a covariate in the analyses. The experiment with nicotine included 38 uninfected and 52 infected bees from three experimental colonies. The experiment with anabasine included 39 uninfected and 40 infected bees from five experimental colonies. Bees from each experimental colony were tested on separate dates.

#### Statistical analysis

Effects of infection and phytochemicals on consumption were analyzed with linear mixed-effects models [70], with solution consumption as the response variable. Infection treatment, phytochemical treatment, and their interaction were used as predictors. Marginal cell length was included as a covariate. Experimental colony and individual bee were included as random effects. We conducted separate analyses for nicotine and anabasine.

### Effects of infection and anabasine on Life History in microcolonies

In a 2 x 2 factorial design, 87 microcolonies were randomly assigned to an infection treatment (uninfected or individually infected with 6000 *Crithidia* cells) and an anabasine treatment (control or 5 ppm anabasine in 30% sucrose) (n = 27 uninfected, control solution; n = 24 uninfected, anabasine solution; n = 16 infected, control solution; n = 20 infected, anabasine solution). Each microcolony consisted of three newly emerged bees, all from the same experimental colony with the same date of eclosion; in total, 10 experimental colonies were used (4 to 15 microcolonies per colony). In this experiment, worker bees were isolated directly from pupal clumps, rather than allowed to emerge in their colonies. The pupal clumps were excised from colonies weekly, incubated at ~27°C in darkness and checked daily for bee emergence. Treatments to bees in the microcolony began following 48 h acclimation to the new nest environment (described in the next paragraph). Each bee was individually infected with 6000 *Crithidia* cells. Uninfected bees were also starved and then fed a sham 10 μL droplet of 25% sucrose solution without *Crithidia* to control for handling.

Microcolonies were housed in 500 mL transparent plastic cylindrical deli containers (see Figure C in S1 File). Each day, microcolonies were fed a ~300 mg piece of pollen paste made of ground pollen (Koppert, Howell, MI) moistened with ~100 μL 30% sucrose. Pollen consumption was estimated by weighing the pollen ball to the nearest 0.0001 g before and after the daily feeding interval. Sucrose solutions were administered via a petri dish (90 mm diameter x 15 mm height), with a 4 cm dental cotton wick in a hole in the dish's cover to allow access. Petri dishes with treatment solutions were replenished daily. Consumption was estimated by weighing the dish and the wick to the nearest 0.001 g before and after each daily feeding interval. Microcolonies were observed daily for worker death, egg production, and honeypot construction.

Microcolonies were terminated 14 d after production of their first egg. This time period was long enough to allow larvae to develop to later instars for measurement of microcolony reproductive output, but short enough to complete the experiment within the lifespan of a typical worker bee [57]. The workers were dissected to measure *Crithidia* infection (see Parasite Variation Experiment: Experimental Inoculation), and bee size (estimated by measurement of the marginal cell of the right forewing). Microcolonies were then frozen for at least 48 hrs and dissected, and all larvae were weighed to the nearest 0.0001 g. If a worker died before the microcolony produced an egg, the microcolony was terminated, and all of its workers were immediately dissected for analysis of infection intensity (see Parasite Variation Experiment: Experimental Inoculation). Microcolonies that neither produced eggs nor incurred worker deaths were followed for up to 30 d, at which point they were recorded as not having laid any eggs, and *Crithidia* infection and bee size were measured in the workers.

#### Statistical analysis

Infection intensity was analyzed as in the Parasite Variation experiment, with anabasine treatment as a fixed predictor, and microcolony nested within experimental colony as random effects to account for non-independence of bees from the same microcolony and colony. Microcolony size dimorphism, defined as [(marginal cell length _largest bee_ / marginal cell length _smallest bee_) - 1], was initially included as a covariate [31], but excluded from the final model because it did not explain significant variation in infection (χ2 = 0.93, df = 1, *p* = 0.33). Due to unbalanced replication across colonies, we did not test for variation in effects of anabasine across colonies. Only experimentally infected bees were included in the analysis of infection intensity; no bees in the uninfected treatment had detectable *Crithidia* infection.

Sucrose and pollen consumption were analyzed by linear mixed models with consumption (g bee^-1^ day^-1^) as the response variable; infection, anabasine, and their interaction as predictors, and microcolony as a random effect. Experimental colony was included as a random effect in the model of pollen consumption, but omitted from the model of sucrose consumption because it did not explain significant variation (χ2 = 0.29, df = 1, *p* = 0.59). Exploratory plots showed that consumption rose and fell over the course of the experiment. To accommodate this pattern, consumption models included both time (days since inoculation) and time^2^ terms as covariates.

Rates of worker mortality, first egg production, and first honeypot construction were tested by Cox mixed-effects proportional hazards models. These analyses used event hazard rate as the response variable; infection, anabasine, and their interaction as predictors; and experimental colony as a random effect. Microcolony size dimorphism, defined as [(marginal cell length _largest bee_ / marginal cell length _smallest bee_) - 1], was included as a covariate in the mortality model, due to its suspected influence on dominance hierarchy within the microcolony [31]. Probability of egg production by the end of the experiment was also analyzed with a binomial model that used infection, anabasine, and their interaction as predictors; and experimental colony as a random effect.

For the subset of 33 microcolonies that produced eggs, production of larvae during the 14 d between first egg production and dissection was analyzed in a gamma family generalized linear mixed model with a log link function. Data were transformed by adding a trivial amount of mass (0.0001 g) to each microcolony’s total before the model was fit. This allowed inclusion of the three microcolonies that produced eggs but no larvae in the gamma family model, which requires positive values of the response variable. Infection treatment, anabasine treatment, and their interaction were used as predictors, and experimental colony as a random effect. Microcolony size dimorphism was initially included, but excluded from the final model because it did not explain significant variation in larval mass.

### Effects of anabasine on infection under pollen deprivation

This experiment tested the effects of *Crithidia* infection and dietary anabasine on infection intensity and sucrose consumption under conditions of pollen deprivation. The *Crithidia* lineage was the same as that used in the Microcolony Experiment. As in the Microcolony Experiment, bees were obtained from pupal clumps that had been excised from the colony. Newly emerged bees were weighed, fed control diets (no anabasine) with pollen for 48 h, then infected 2 d post-emergence using 25% sucrose with or without 6000 *Crithidia* parasite cells. Thereafter, bees were maintained in individual vials for 7 d (28°C, constant darkness). Bees were moved to clean vials daily, at which time we provided a clean feeder lid that contained 500 μL sucrose solution either with or without 5 ppm anabasine. To estimate sucrose consumption, the feeder lids were weighed before and after each 24 h consumption interval. Deaths were recorded daily. Bees that survived the 7 d experiment were dissected to measure infection intensity. The experiment included 182 bees (n = 45–47 bees per treatment combination).

#### Statistical analysis

Infection was analyzed with ln (x+1)-transformed *Crithidia* count as the dependent variable, anabasine treatment as a fixed effect; bee mass at emergence as a covariate; and inoculation dates as random effects. However, in this experiment we had sufficiently balanced samples to include colony as a fixed effect to test the anabasine by colony interaction term, to evaluate whether the effects of anabasine varied across experimental colonies. Sucrose consumption was analyzed with infection, anabasine, and their interactions as fixed effects; experimental colony, individual bee, and measurement date as a random effect, and bee mass at emergence as a covariate.

## Results

### *Crithidia* infection

In all experiments, *Crithidia* inoculation was successful, with high rates of parasite replication. Median control treatment infection levels were 255 * 10^3^ (Parasite Variation), 180 * 10^3^ (Life History), and 247.5 * 10^3^ (Pollen Deprivation) *Crithidia* cells bee^-1^; these values represent 30- to 44-fold increases over the 6 * 10^3^ cells with which bees were inoculated.

**Fig 1:**
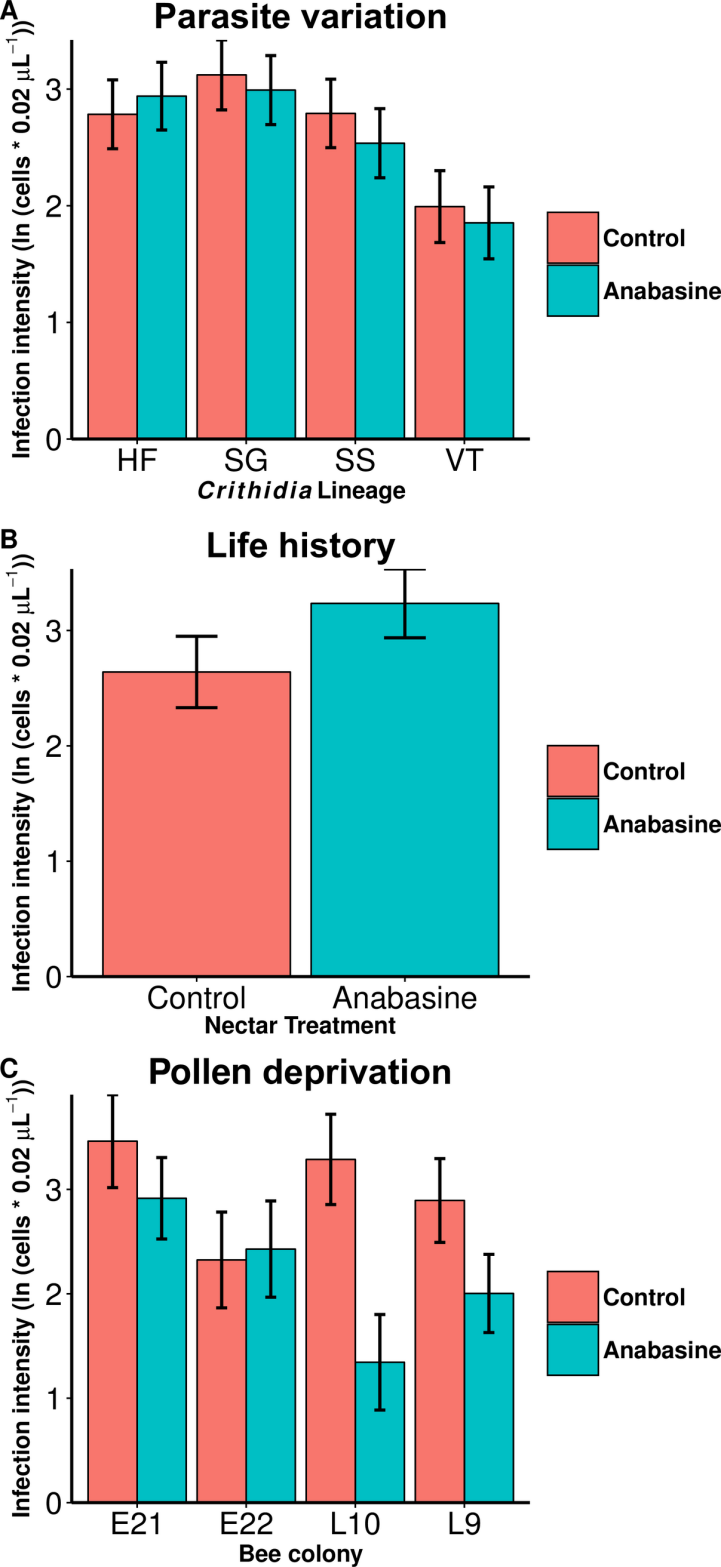
Variable effects of anabasine treatment on *Crithidia* infection intensity in *Bombus impatiens*. Y-axis shows least squares means and standard errors for ln(x+1)-transformed parasite count in 0.02 μL gut extract. Colors denote diet treatments (30% sucrose control and 5 ppm anabasine in 30% sucrose). (A) Parasite Variation: Anabasine did not reduce infection with any of four *Crithidia* lineages from different locations. Samples sizes: n = 49–70 bees for each combination of lineage and diet treatment (see Table A in S1 File for exact sample sizes). HF: Hampshire Farm, Amherst, MA. SG: Simple Gifts Farm, Amherst, MA. SS: Stone Soup Farm, Amherst, MA. VT: Route 5, Norwich, Vermont, USA. (B) Life History Experiment: Anabasine did not reduce infection in microcolonies. Sample size: n = 29 bees per treatment. (C) Pollen Deprivation Experiment: In two colonies, anabasine reduced infection in bees deprived of pollen. The effects of anabasine varied across colonies (Table 1). Sample sizes: n = 3–11 bees per colony and treatment, 72 bees total.

Anabasine generally did not reduce infection intensity, with the exception of two colonies in the Pollen Deprivation Experiment. In the Parasite Variation Experiment, which was the largest of the three that assessed infection, the parasite lineages varied in infectivity, with 59% higher log-transformed infection intensity in the most infective lineage, SG, than in the least infective lineage, VT. However, there was no effect of anabasine ingestion on infection intensity for any of the lineages (Fig 1A, Table 1). Similarly, anabasine had no effect on infection in the Life History Experiment (Fig 1B, Table 1), where anabasine nonsignificantly increased infection intensity. However, median parasite count was more than twice as high in anabasine-fed bees as in controls (25 vs 12 cells per 0.02 μL gut homogenate). In contrast, anabasine did reduce infection intensity in the Pollen Deprivation Experiment by 27% overall (Fig 1C, Table 1). However, this effect was driven by two of the four colonies, as indicated by the significant anabasine by colony interaction (Table 1). Anabasine reduced infection in colonies L10 (t = -3.82, *p <* 0.001) and L9 (t = -2.07, *p* = 0.044), but not in colonies E21 (*p* = 0.27) or E22 (*p* = 0.85). In contrast, there was no significant anabasine by colony interaction in the Parasite Variation Experiment (*p* > 0.15), and insufficient replication to test this interaction in the Life History Experiment.

**Table 1:** Effects of 5 ppm anabasine treatment on *Crithidia* infection intensity in *Bombus impatiens* across three experiments. (A) Parasite Variation Experiment that tested effects of anabasine on infection of individual bees with one of four *Crithidia* lineages and reared individually. (B) Life History Experiment in which bees were reared in microcolonies of three workers. (C) Pollen Deprivation Experiment in which individual bees were deprived of pollen. Significance of terms in generalized linear mixed-effects models were tested by χ^2^ tests. *Crithidia* cell counts were ln(x+1)-transformed to better conform to model assumptions. Marginal cell length refers to length of the right forewing marginal cell, used to estimate bee size (see Materials and Methods). Colony refers to the bee’s experimental colony of origin.

**A. Parasite Variation Experiment**	**χ**^**2**^	**df**	**P**
Anabasine	0.44	1	0.51
Lineage	11.69	3	**0.009**
Mass	13.92	1	**<0.001**
Anabasine by Lineage	1.46	3	0.69
**B. Life History Experiment**	**χ**^**2**^	**df**	**P**
Anabasine	1.92	1	0.17
Marginal cell length	2.37	1	0.12
**C. Pollen Deprivation Experiment**	**χ**^**2**^	**df**	**P**
Anabasine	11.82	1	**<0.001**
Colony	7.39	3	0.061
Anabasine by Colony	7.97	3	**0.047**

### Food consumption

**Fig 2:**
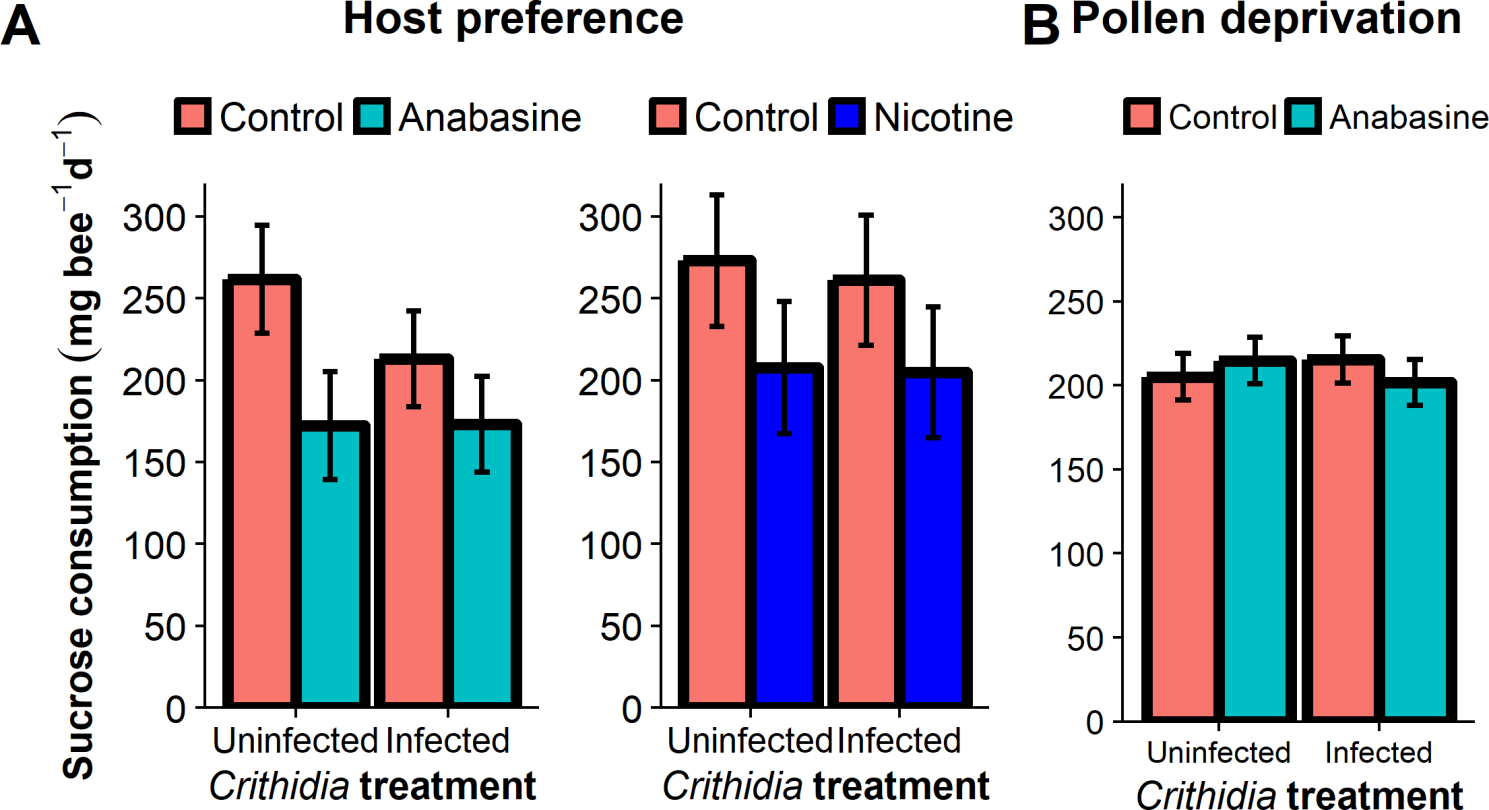
**Effects of *Crithidia* infection and 5 ppm anabasine or 2 ppm nicotine on consumption of sucrose solutions and pollen in choice (A) and no-choice (B) experiments.** (A) Host Preference Experiments, in which individual bees were given a choice between 30% sucrose solutions with and without 5 ppm anabasine or 2 ppm nicotine. The 24 h trial was conducted 7 d post-infection. (B) Pollen Deprivation Experiment solution consumption by individual bees under no-choice conditions. Consumption was measured over the entire experiment (7 d). Bars and error bars represent model means and standard errors. Red bars: 30% sucrose control. Teal bars: 5 ppm anabasine. Blue bars: 2 ppm nicotine.

We did not find evidence for infection-induced alkaloid preference. In the Host Preference Experiment, alkaloid-free solutions were significantly preferred to nicotine- and anabasine-containing solutions regardless of infection (Table 2). Results were similar for 5 ppm anabasine (higher consumption of control solution by 90 mg bee^-1^ among uninfected bees and 40 mg bee^-1^ among infected bees); and for 2 ppm nicotine (higher consumption of control solution by 65 mg bee^-1^ among uninfected and 56 mg bee^-1^ among infected bees). For neither compound did infection alter relative preference (Fig 2A), as indicated by the non-significant infection by phytochemical interaction term in each model (Table 2).

**Table 2:** Effects of *Crithidia* infection and 5 ppm anabasine on consumption of sucrose solution and pollen in choice and no-choice experiments. (A) In Host Preference Experiments, individual bees were given a choice between 30% sucrose solution with and without 5 ppm anabasine. The 24 h trial was conducted 7 d post-infection. (B) Consumption in microcolonies of Life History Experiment and (C) of individual bees in Pollen Deprivation Experiment under no-choice conditions. Consumption was measured over the entire experiment (up to 35 d for Life History Experiment, 7 d for Pollen Deprivation Experiment). Time was not a significant predictor of consumption in the Pollen Deprivation Experiment.

**A. Host Preference Experiments**			
**Nicotine (2 ppm)**	**χ**^**2**^	**df**	**P**
Infection	0.12	1	0.72
Nicotine	8.34	1	**0.004**
Marginal cell length	15.14	1	**<0.001**
Infection by Nicotine	0.04	1	0.84
**Anabasine (5 ppm)**	**χ**^**2**^	**df**	**P**
Infection	0.98	1	0.32
Anabasine	6.83	1	**0.009**
Marginal cell length	16.90	1	**<0.001**
Infection by Anabasine	1.14	1	0.29
**B. Life History Experiment**			
**Sucrose consumption**	**χ**^**2**^	**df**	**P**
Infection	2.42	1	0.12
Anabasine	0.11	1	0.74
Time	225.46	1	**<0.001**
Time^2^	176.53	1	**<0.001**
Infection by Anabasine	0.85	1	0.36
Infection by Anabasine by Time	5.93	3	0.12
**Pollen consumption**			
Infection	5.86	1	0.015
Anabasine	0.01	1	0.92
Time	340.57	1	**<0.001**
Time^2^	175.98	1	**<0.001**
Infection by Anabasine	1.52	1	0.22
**C. Pollen Deprivation Experiment**	**χ**^**2**^	**df**	**P**
Infection	1.47	1	0.22
Anabasine	1.33	1	0.25
Mass	11.13	1	**<0.001**
Infection by Anabasine	3.85	1	**0.0497**

In the Life History Experiment, neither infection nor anabasine treatment affected sucrose consumption (Table 2, Figure D in S1 File), which initially rose, then fell over time in a similar fashion across treatments (Fig 2B). Infection significantly reduced pollen consumption by 0.99 mg bee^-1^ d^-1^ (Table 2, Figure D in S1 File), but anabasine treatment had no effect on pollen consumption (Table 2).

For individual bees in the Pollen Deprivation Experiment, there was a significant infection by anabasine interaction in the opposite direction of what was predicted based on the hypothesis of self-medication (Table 2). Among uninfected bees, we found higher consumption of 5 ppm anabasine than control solution by 9.5 mg bee^-1^ day^-1^, whereas among infected bees, we found lower consumption of 5 ppm anabasine than control solution by 13.5 mg bee^-1^ day^-1^ (Fig 2B). There were no significant anabasine by colony (*p* = 0.58) or infection by anabasine by colony (*p* = 0.85) interactions.

### Survival and performance

**Fig 3:**
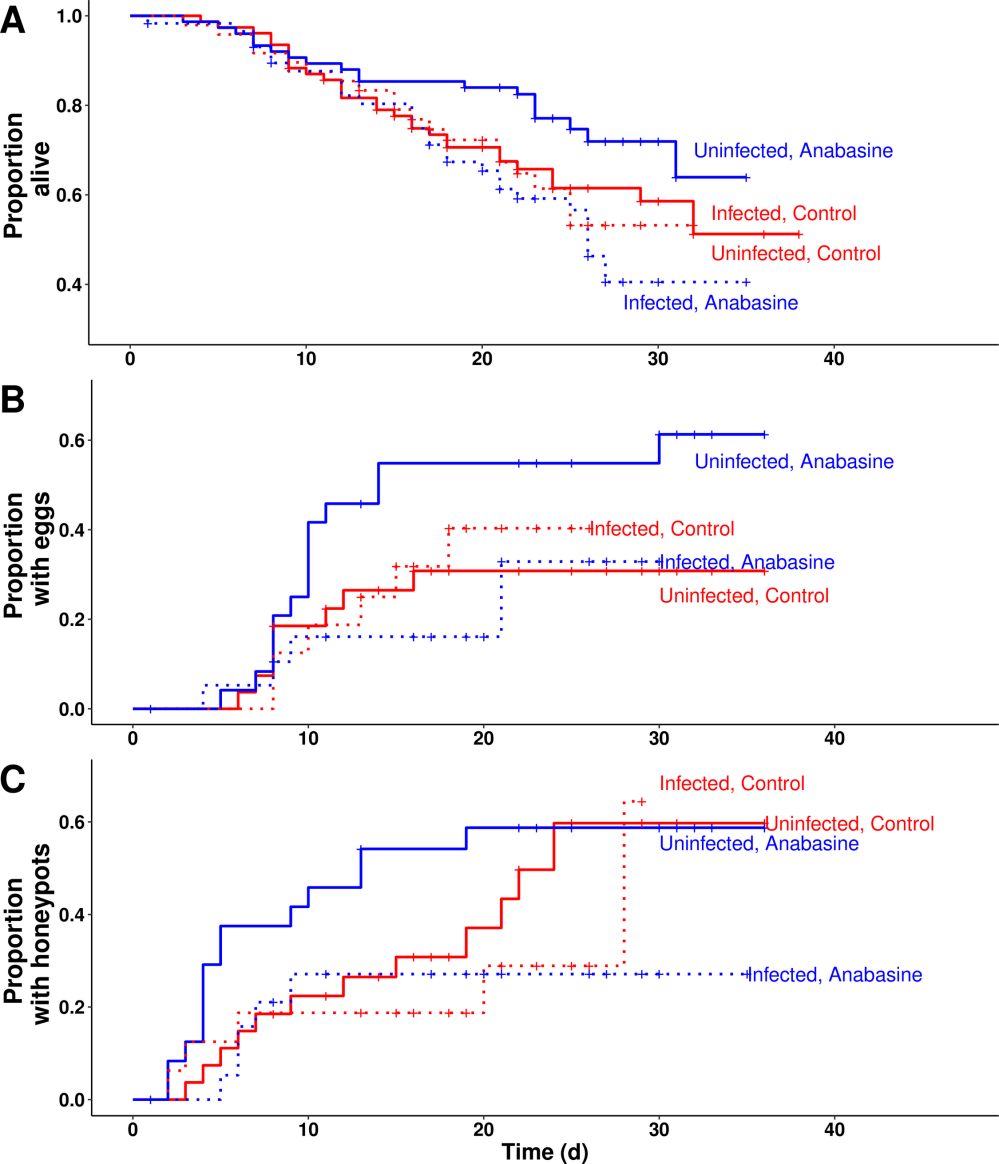
Effects of *Crithidia* infection on microcolony performance in the Life History Experiment. Microcolonies were observed daily for (A) deaths, (B) time to first egg production, and (C) time to first honeypot construction. Line type represents infection treatment (solid lines for uninfected microcolonies; dotted lines for infected microcolonies). Line color represents anabasine treatment (red lines for 30% sucrose control; blue lines for 30% sucrose with 5 ppm anabasine). Crosses represent events (i.e., deaths, egg production, or honeypot construction) or censoring due to removal of the microcolony from the experiment.

**Fig 4:**
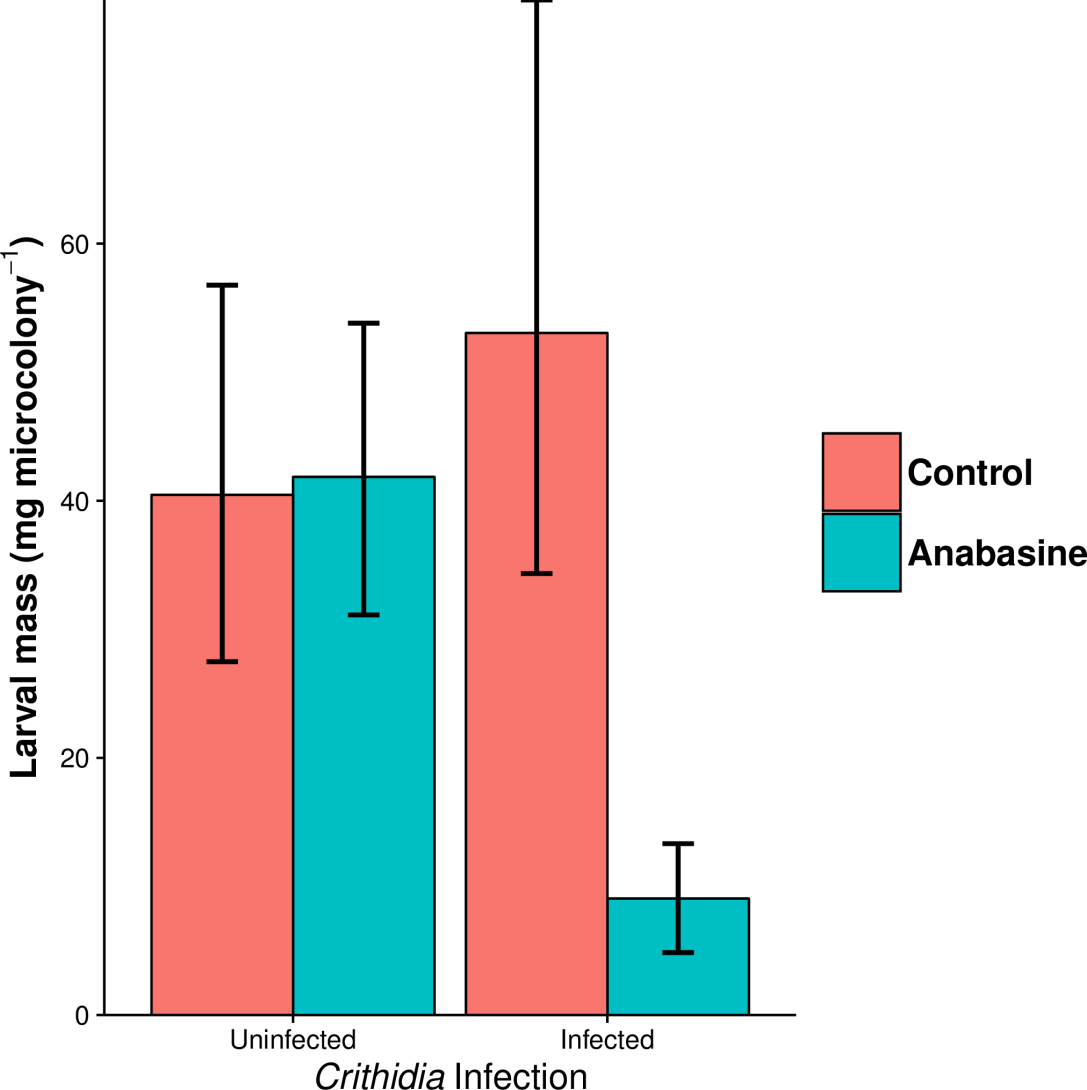
Effects of *Crithidia* infection on microcolony production of larvae in the Life History Experiment. Microcolonies that produced eggs were dissected 14 d after first egg production for measurement of larval masses. **Bars and error bars show least squares means and standard errors. Error bars are asymmetric due to back-transformation via the gamma family model’s log link function.**

In the Life History Experiment, we observed significant interactive effects of infection and anabasine on bee health, such that anabasine had neutral or beneficial effects on uninfected bees, but deleterious effects on infected bees. In particular, anabasine increased survival among uninfected but decreased survival among infected bees (Fig 3A; Table 3A). Effects of infection and anabasine on time to first egg production were not statistically significant (Fig 3B, Table 3B). However, in a binomial model that considered only endpoint probability of egg production, the infection by anabasine interaction (χ^2^ = 4.23, df = 1, *P* = 0.040) was significant. Anabasine nearly doubled the probability of egg production among uninfected bees (58% vs 30%, *post hoc* pairwise comparison: Z = 2.27, *P* = 0.023), but resulted in a non-significant decrease in the probability of egg production among infected bees (25% vs 38%, Z = -0.79, *P* = 0.43). Time to first honeypot production was significantly affected by infection but not anabasine (Table 3C). Over the first four weeks of the experiment, rates of first honeypot formation were similarly depressed in infected bees regardless of anabasine treatment (Fig 3C). Anabasine also had strong deleterious effects on larval production in infected microcolonies. Anabasine had a negative main effect on larval mass that was driven by its effects in infected microcolonies, where total larval mass was reduced more than 80% relative to all other treatment groups (Fig 4). The infection by anabasine interaction was statistically significant (Table 3D). Together, these results indicate that anabasine aggravated the effects of infection on colony productivity.

**Table 3:** Effects of *Crithidia* infection and anabasine on microcolony performance in the Life History Experiment. Microcolonies were observed daily for (A) survival of individuals, (B) time to first egg production, and (C) time to first honeypot construction. Differences in hazard rates across treatments were assessed by Cox mixed-effects proportional hazards models. (D) Total larval mass per microcolony at 14 d after first egg production was tested for the subset of microcolonies that produced eggs.

**A. Survival**	**χ2**	**df**	**P**
Infection	3.72	1	0.054
Anabasine	0.64	1	0.43
Size dimorphism	3.74	1	0.053
Infection by Anabasine	3.86	1	**0.0495**
**B. Egg production**	**χ2**	**df**	**P**
Infection	1.06	1	0.30
Anabasine	1.59	1	0.21
Infection by Anabasine	2.60	1	0.11
**C. Honeypot construction**	**χ2**	**df**	**P**
Infection	3.96	1	**0.047**
Anabasine	0.91	1	0.34
Infection by Anabasine	0.54	1	0.46
**D. Larval mass**	**χ2**	**df**	**P**
Infection	2.78	1	0.095
Anabasine	5.28	1	**0.022**
Infection by Anabasine	5.70	1	**0.017**

Little mortality was observed in the Parasite Variation (46 deaths among 602 total bees) and Pollen Deprivation Experiments (34 deaths among 182 bees), both of which lasted only 7 d. Mortality negatively covaried with mass in the Parasite Variation Experiment (chi-squared = 12.88, df = 1, *p* <0.001), such that larger bees had a reduced risk of death (z = -3.59). However, anabasine did not affect mortality in the Parasite Variation Experiment (chi-squared = 0.19, df = 1, *p* = 0.66), and neither infection treatment (chi-squared = 0.82, df = 1, *p* = 0.37), anabasine treatment (chi-squared = 1.47, df = 1, *p* = 0.23), nor their interaction (chi-squared = 0.52, df = 1, *p* = 0.47) affected survival in the Pollen Deprivation Experiment (see Figure E in S1 File for survival curves).

## Discussion

We tested the effects of naturally occurring concentrations of the nectar alkaloid anabasine on bumble bee infection with multiple parasite lineages of *Crithidia*, examined whether infection altered phytochemical preference, and assessed phytochemicals’ effects on performance of infected versus uninfected bees. Our results did not support any of the four hypotheses that motivated the study. Anabasine had medicinal effects but only in one study under pollen-starved conditions; infection did not result in preference for anabasine; and anabasine was relatively innocuous for uninfected bees but deleterious for infected bees. We discuss these results in the context of our four motivating hypotheses.

### Hypothesis 1. Anabasine consumption generally reduces infection, but medicinal effects vary due to differences in sensitivity among parasite lineages

We tested effects of anabasine on infection with four distinct parasite lineages. Although these lineages were phenotypically distinct in terms of overall infectivity, anabasine was not effective against infection with any of the lineages. All our lineages were collected within New England, and three of four came from within a single town. This area does not encompass the broad geographic distribution of *Crithidia*, which is found in North America, Europe, and South America [79,80]. Thus, we cannot rule out the possibility that anabasine has effects on *Crithidia* collected from other parts of its range. However, we should note that one of the studies that found medicinal effects of anabasine on *Crithidia* used a lineage collected in the same region [16]. The contrast between the lack of antiparasitic effects shown here with the presence of antiparasitic effects shown previously provides further evidence for variability in anabasine’s effects on *Crithidia* infection, but provides no evidence that differences between parasite lineages are responsible for this variation.

Our results cast doubt on the hypothesis that variation in the effects of anabasine on *Crithidia* are due to direct effects of this compound on parasite fitness. If between-strain variation were the explanation for this variation, then the effects of phytochemicals should at least be consistent within a *Crithidia* lineage. However, anabasine did not reduce infection of microcolonies in the Life History Experiment, but did reduce infection on two colonies under Pollen Deprivation, even though these two experiments were conducted with the same *Crithidia* lineage. Although we cannot rule out that a lineage may change over time, in *vitro* experiments suggest that *Crithidia* are quite robust to nicotine and anabasine. For example, concentrations of >500 ppm of nicotine or anabasine were necessary for *Crithidia* growth inhibition [21,81], which are drastically higher concentrations than the <10 ppm found in *Nicotiana* nectar [13,59,66].

Rather than direct effects on parasites, anabasine’s effects on infection may reflect the compound’s effects on bees, which do have demonstrated sensitivity to nicotine and other alkaloids [24]. Nicotine, anabasine, and neonicotinoids are all agonists of nicotinic acetylcholine receptors, which are abundant in insect brains [82–85]. Indeed, nicotine has inspired an entire class of synthetic neonicotinoid insecticides that specifically target the acetylcholine receptors of insects [86]. All of these compounds are stimulants at low concentrations, but cause spasms, paralysis, and death at higher levels [24,87]. It is possible that anabasine provokes intestinal spasms that interfere with parasite attachment to the gut wall [88], or lead to midgut carbohydrate malabsorption that increases the sugar content in the distal gut lumen, and thereby exposes hindgut parasites like *Crithidia* to osmotic stress that inhibits growth [89]. Our uninfected, *ad libitum* fed microcolonies did not appear harmed by, and in some cases benefited from anabasine. However, wild bees under more stressful conditions, including infection, might be less robust to potential costs of anabasine ingestion.

### Hypothesis 2. Bees self-medicate, such that relative preference for phytochemicals increases under conditions of infection

In the Host Preference, Life History, and Pollen Deprivation experiments, we tested whether infection would alter preference for anabasine and, in the Host Preference Experiment, nicotine. In the Host Preference Experiment, when bees were given a choice between phytochemical-containing and phytochemical-free solutions, the phytochemical-free solution was preferred regardless of infection status. These findings contrast with prior work, in which infected *Bombus terrestris* workers visited nicotine-containing artificial flowers with relatively high frequency, whereas uninfected bees had no preference [15]. It is possible that bees do visit nicotine-containing flowers with greater frequency when infected, but that they consume less nectar at each visit [90]; that preferences of free-flying bees differ from those of caged bees; or that aversion to nicotine differs in *B*. *impatiens* and *B*. *terrestris*. We also cannot exclude the possibility that preferences may differ at time points other than 7 d post-infection. Our results suggest that *B*. *impatiens* and *B*. *terrestris* may be more sensitive to nicotine than is *Apis mellifera*, which displayed no aversion to 2 ppm nicotine [26], and had a deterrence threshold of 300 ppm [24]. *B*. *terrestris* total sugar water consumption was likewise more suppressed by availability of neonicotinoids than was *Apis mellifera* consumption [91]. The observation that anabasine is generally deterrent for both honey bees [26] and bumble bees (our results) indicates that, even if anabasine does counteract infection, the deterrent effects of anabasine and nicotine may limit the influence of these compounds, at least in bee populations that have access to anabasine-free flowers.

Our no-choice experiments provide additional evidence against infection-dependent benefits of anabasine. If bees self-medicate with anabasine, the deterrent effects of anabasine should be weaker when bees are infected. However, the Life History Experiment found no effect of infection on anabasine consumption. Furthermore, the Pollen Deprivation Experiment found the opposite pattern of what was predicted under the hypothesis of self-medication: anabasine consumption was highest in *uninfected* bees, whereas infected bees consumed less anabasine solution than control solution. These results contrast with prior work in which 20 ppm anabasine increased sucrose consumption in both infected and uninfected *B*. *impatiens* microcolonies under no-choice conditions [16]. Although it would be most parsimonious to assume that the effects of anabasine are unidirectional, it is possible that higher concentrations elicit greater consumption, possibly due to stimulatory effects that increase energetic requirements. Experiments that employ a range of concentrations would be needed to test this hypothesis. Still, given that even low doses (2.5 ppm) of nicotine can elevate bumble bee mortality [15], and that both nicotine and anabasine have inconsistent effects against parasite infection [17,18], it seems unlikely that preference for these compounds would be favored in host populations.

### Hypothesis 3. Phytochemical consumption is deleterious for uninfected bees, but beneficial for infected bees

If self-medication is a form of adaptive plasticity, then phytochemical consumption should be neutral or harmful to uninfected individuals, but beneficial to infected individuals [12]. In contrast, we found that anabasine had neutral or positive effects on uninfected bees, but negative effects when combined with infection. In microcolonies of our Life History Experiment, anabasine treatment showed no significant effect against parasites, and in fact doubled median infection intensity (25 vs. 12 *Crithidia* cells * 0.02 μL^-1^), a difference that is striking, but was not statistically significant. In terms of performance, anabasine did not affect survival, time to egg production, time to honeypot construction, or larval production by uninfected bees. We did find significant interactive effects of infection and anabasine on survival and larval production, such that the combination of infection and anabasine treatment had negative effects of these fitness correlates (Fig 3, Table 3). Our results contrast with previous reports, in which 20 ppm anabasine consumption reduced survival in uninfected bees but improved survival in infected bees [16].

However, in the context of other experiments, our results suggest that chemical sensitivity of both bumble bees and honey bees may be exacerbated by infection. In uninfected bees, the 48 h nicotine LD50 in *Apis mellifera* was 2000 ppm, up to 50 ppm had no effects on forager mortality [65], and up to 5 ppm did not affect honey production. However, in immune-challenged honey bees, a mere 0.5 ppm nicotine increased mortality [92]. Similarly, the effects of neonicotinoid insecticides on bee mortality were exacerbated by concurrent *Crithidia* [93] and *Nosema* infection [94,95]. Neonicotinoids are, like anabasine and nicotine, acetylcholine receptor agonists, so it is logical that each of these compounds would have similar effects on bees. Chemical exposure may directly suppress immunity [94,96], and the combined stresses of chemical exposure and infection may result in trade-offs between immunity and detoxification, both of which are energetically expensive [38,97].

Although effects of phytochemicals on colony-level fitness cannot be fully understood from experiments on isolated workers, workers are needed to gather and distribute resources, and their ability to do so may be compromised by infection [49], acetylcholine receptor agonists [98], and mortality. Workers may also transmit infection to other colony members [99], including new queens, and thereby reduce success of colonies the following year [36]. We cannot rule out that other phytochemical concentrations might have stronger benefits against infection or lesser costs than those observed here, or that benefits are restricted to particular levels of infection. However, our experiments suggest that anabasine and infection may exacerbate one another’s effects.

### Hypothesis 4. Phytochemical consumption has the greatest benefits in well-nourished hosts, which are less susceptible to phytochemical-induced toxicity

In contrast to our hypothesis, anabasine consumption only reduced infection intensity in two colonies of bees that were deprived of pollen post-infection. There are several possible explanations for this effect. One possibility is that the parasite lineage used in this experiment was more susceptible to anabasine than those used in other experiments. However, this was the same lineage used in the Life History Experiment, in which no antiparasitic effects of anabasine were found. A second possibility is that anabasine only affects parasites that are already weakened by nutritional stress due to pollen deprivation, which has been shown to reduce infection levels [40,42]. Our data do not support this hypothesis, either. Overall infection intensity (*Crithidia* cell concentration) was at least as high in the Pollen Deprivation Experiment as in other experiments (Fig 1), although we cannot rule out that *Crithidia* cells may have been less robust to chemicals in pollen-starved bees.

A third possibility is that the effect of anabasine depends on genetic variation in bees and their associated gut microbiota. Anabasine and other nAchR’s have strong excitatory effects on insect brain neurons, where acetylcholine is the primary excitatory neurotransmitter [84,85]. These excitatory effects may raise the frequency or intensity of defensive defecation that occurs when bees are startled. This behavior might expel parasites from the hindgut. If bee genotypes vary in sensitivity to anabasine, in the same way that diverse insect species vary in sensitivity to nicotine [60], bees of some colonies might be more strongly affected by the compound. This idea is supported by the significant variation in anabasine’s medicinal effects across colonies. It could be that prior experiments that found medicinal effects of anabasine [16,100] used colonies that happened to be exceptionally sensitive to this compound, harbored microbiota with low rates of anabasine catabolism, or converted anabasine into compounds with stronger antiparasitic effects.

Two factors may have magnified the effects of anabasine in the Pollen Deprivaiton Experiment. The first is the absence of protein from dietary pollen. Pollen consumption is necessary for induction of detoxification genes [37]; absence of pollen may lead to stronger effects of alkaloids due to inability to metabolize these chemicals. This idea is supported by the reduced nicotine tolerance observed in honeybees fed low-protein diets [101]. Pollen would also have provided bees an alternative, anabasine-free food source, which could have decreased total consumption of anabasine. We note that all the experiments to date that have shown medicinal effects of anabasine included factors that promoted high anabasine intake, particularly during the initial stages of infection. For example, whereas we starved bees for 2–3 h pre-inoculation, Richardson *et al*. [16] and Anthony *et al*. [100] starved bees overnight, which may have resulted in high anabasine consumption immediately following infection as bees attempted to refeed and rehydrate. Similarly, another trial that showed medicinal effects of anabasine [18] was conducted under hot and variable conditions that may have raised carbohydrate and water requirements and prompted higher consumption of anabasine-containing solutions when this was the only liquid available. Specifics of each of these prior experiments are summarized in S1 Appendix.

Second, bees in the Pollen Deprivation Experiment were taken directly from pupal clumps that had been excised from the rest of the colony, and had no opportunity to acquire microbiota from nest mates. This horizontal transmission is necessary to colonize the relatively sterile gut of newly emerged bees [102]. Although experimental bees were inoculated with whole-gut homogenates at the time of infection, a lack of pre-colonization with core microbiota may have exacerbated susceptibility to infection [103], and thereby made it easier to discern medicinal effects of phytochemicals. In addition, if microbiota themselves can metabolize phytochemicals [104,105], lack of symbiotic microbiota may have reduced the rate of anabasine breakdown, and thereby increased the potency of the ingested dose. Although bees in the Life History Experiment were also taken from pupal clumps, infection in these bees was not assessed for several weeks after inoculation with *Crithidia-*containing gut homogenates. This longer period may have allowed more time for core gut microbiota to establish and modulate anabasine metabolism over the course of the experiment. The role of the bee gut microbiome in modulation of antiparasitic effects of phytochemicals requires further investigation.

## Conclusions

Our goal was to explore how several factors contribute to variable effects of anabasine on *Crithidia* infection in bumble bees. We found inconsistent evidence for medicinal effects, which may be modulated by host genotype and food environment, and suggest that effects of anabasine on infection intensity are more likely to reflect anabasine’s effects on insects rather than direct effects on the parasite. Alkaloids were deterrent in preference trials regardless of infection status; the absence of infection-induced preference may reflect uncertain benefits of such behavior, in contrast to demonstrated negative effects of anabasine and other acetylcholine receptor agonists on insect survival and fitness.

Although uninfected bees in our experiments were not adversely affected by alkaloid-containing diets, anabasine had deleterious effects on infected bees. This is the first report of exacerbation of floral alkaloids’ negative effects by *Crithidia* infection. This exacerbation is consistent with a growing body of work that suggests the negative effects of combined stressors—including infection, diet quality, and consumption of pesticides—on pollinator health [44], and that widespread infection could have consequences for bees’ ability to tolerate phytochemicals. The reasons for variable effects of nectar phytochemicals on parasite infection warrant further investigation. Especially needed are factorial experiments that test multiple environmental factors and phytochemical concentrations simultaneously. Knowledge of the environmental factors that determine beneficial vs. detrimental effects of phytochemicals on bee health will help explain the ecological implications of phytochemical occurrence, and how these compounds can be applied in pollinator conservation and management.

## Supporting information

S1 Appendix Summary of our own and prior experiments that tested effects of anabasine on bumble bee infection. (XLSX)

S1 Data Raw data for the parasite variation, life history, host preference, and pollen deprivation experiments. (XLSX)

S1 File Supplementary information that includes supplementary tables and figures, and key to S1 data. (DOCX)
