# Supplementary material for: Context-dependent medicinal effects of anabasine and infection-dependent toxicity in bumble bees
Source: PLoS One. 2017 Aug 23;12(8):e0183729. doi: 10.1371/journal.pone.0183729 (PMC5568382; doi:10.1371/journal.pone.0183729)
Supplement: S1 File — (DOCX) [file pone.0183729.s003.docx]

S1 File (Supplementary Information)

Contents

[Supplementary Tables 2](#_Toc490241529)

[Table A. Sample sizes for analysis of infection intensity. 2](#_Toc490241530)

[Supplementary Figures 3](#_Toc490241531)

[Figure A. Rearing environment for individual-bee experiments. 3](#_Toc490241532)

[Figure B. Materials for Host Preference Experiments. 4](#_Toc490241533)

[Figure C. Rearing environment for Life History Experiment. 5](#_Toc490241534)

[Figure D. Effects of anabasine on microcolony consumption of nectar and pollen in the Life History experiment. 8](#_Toc490241535)

[1. Consumption of sucrose solution. 8](#_Toc490241536)

[2. Consumption of pollen. 9](#_Toc490241537)

[Figure E. Survival curves for individual bees 10](#_Toc490241538)

[1. Parasite variation experiment. 10](#_Toc490241539)

[2. Pollen deprivation Experiment 11](#_Toc490241540)

[Supplementary data files (key) 12](#_Toc490241541)

# Supplementary Tables

## **Table A.** Sample sizes for analysis of infection intensity.

1. Parasite variation

| Treatment | Lineage | N |
| --- | --- | --- |
| Control | HF | 60 |
| Anabasine | HF | 67 |
| Control | SG | 60 |
| Anabasine | SG | 70 |
| Control | SS | 55 |
| Anabasine | SS | 55 |
| Control | VT | 50 |
| Anabasine | VT | 49 |

1. Life history (Microcolony) experiment

| Treatment | N |
| --- | --- |
| Control | 29 |
| Anabasine | 29 |

1. Pollen deprivation experiment

| Treatment | Colony | N |
| --- | --- | --- |
| Control | E19 | 5 |
| Anabasine | E19 | 3 |
| Control | E21 | 7 |
| Anabasine | E21 | 9 |
| Control | E22 | 7 |
| Anabasine | E22 | 6 |
| Control | L10 | 8 |
| Anabasine | L10 | 7 |
| Control | L9 | 9 |
| Anabasine | L9 | 11 |

# Supplementary Figures

**Figure A.** Rearing environment for individual-bee experiments.

The 2 mL microcentrifuge was filled with 500 µL of aqueous 30% sucrose, plugged with a dental cotton wick, and inverted to allow the solution to saturate the wick. Vials were incubated horizontally, with the long axes of the tube and vial oriented parallel to the incubation surface. Bees were moved to new vials and solutions replenished daily.


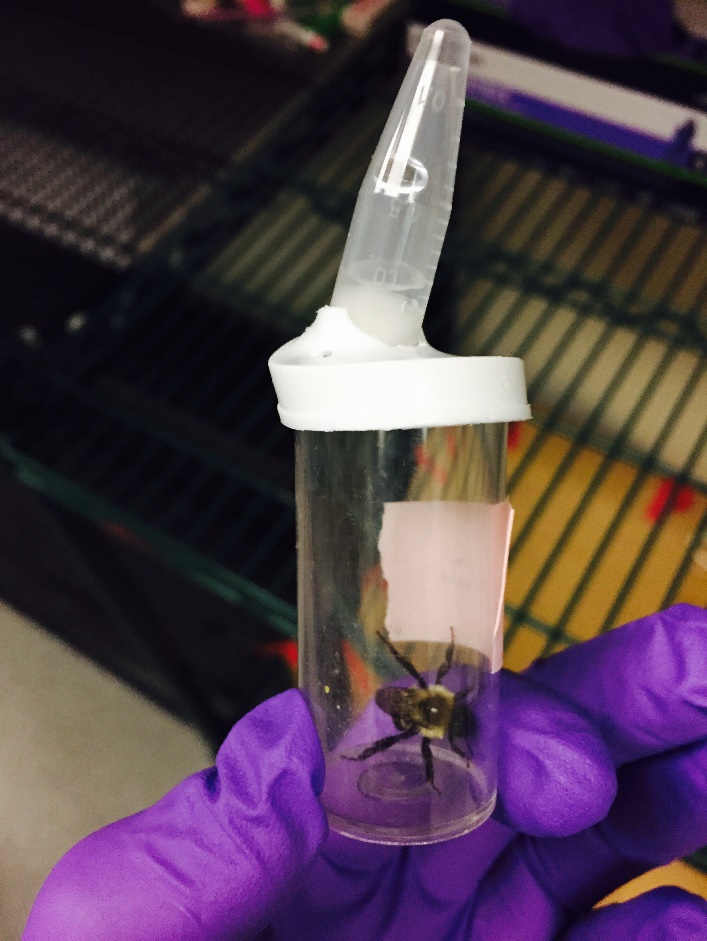


## **Figure B.** Materials for Host Preference Experiments.

(1) Drilled Tube. Black dots indicate locations where feeding holes (2.7 mm diameter) were drilled.


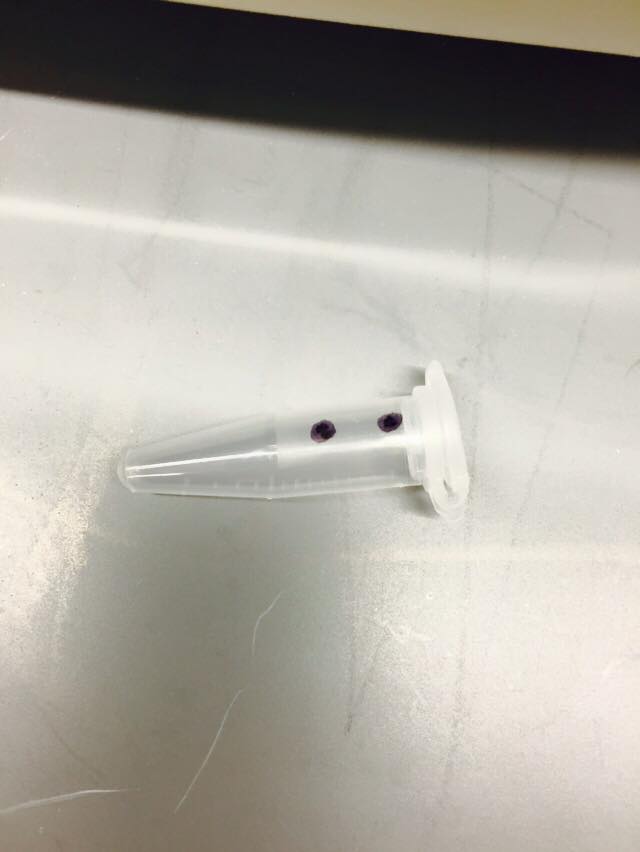


(2) Arena with drilled tubes


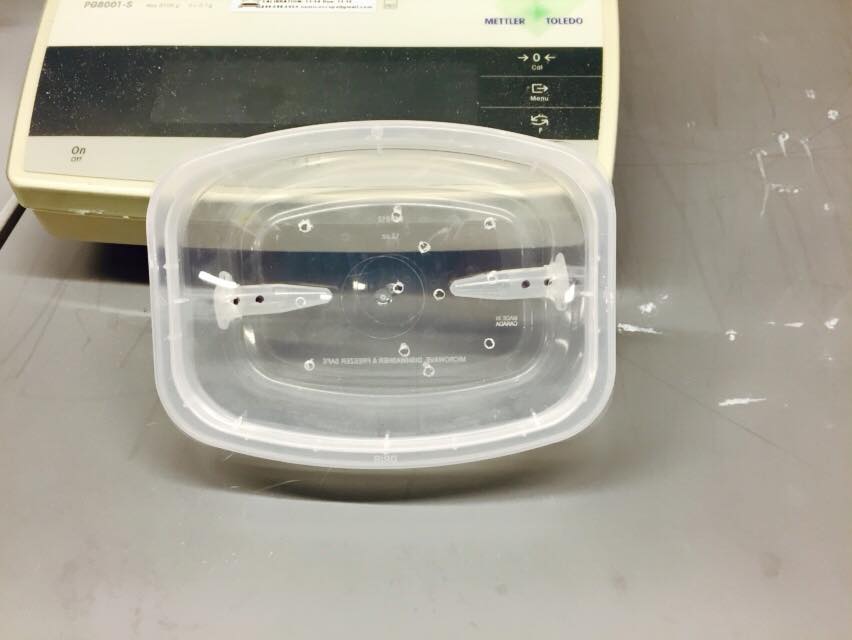


## **Figure C.** Rearing environment for Life History Experiment.

Solutions were administered via a petri dish (90 mm diameter x 15 mm height). The bottom of the microcolony's plastic container was cut off and replaced with wire mesh. The petri dish was filled with the appropriate treatment solution with a 4 cm dental cotton wick in a hole in the dish's cover. The microcolony container was placed on top of the wick. For stability, both the petri dish and the microcolony's container were nested inside a second 500 mL container.

1. The inner cup, which contained the bees. The bottom was removed and replaced with wire mesh.

**
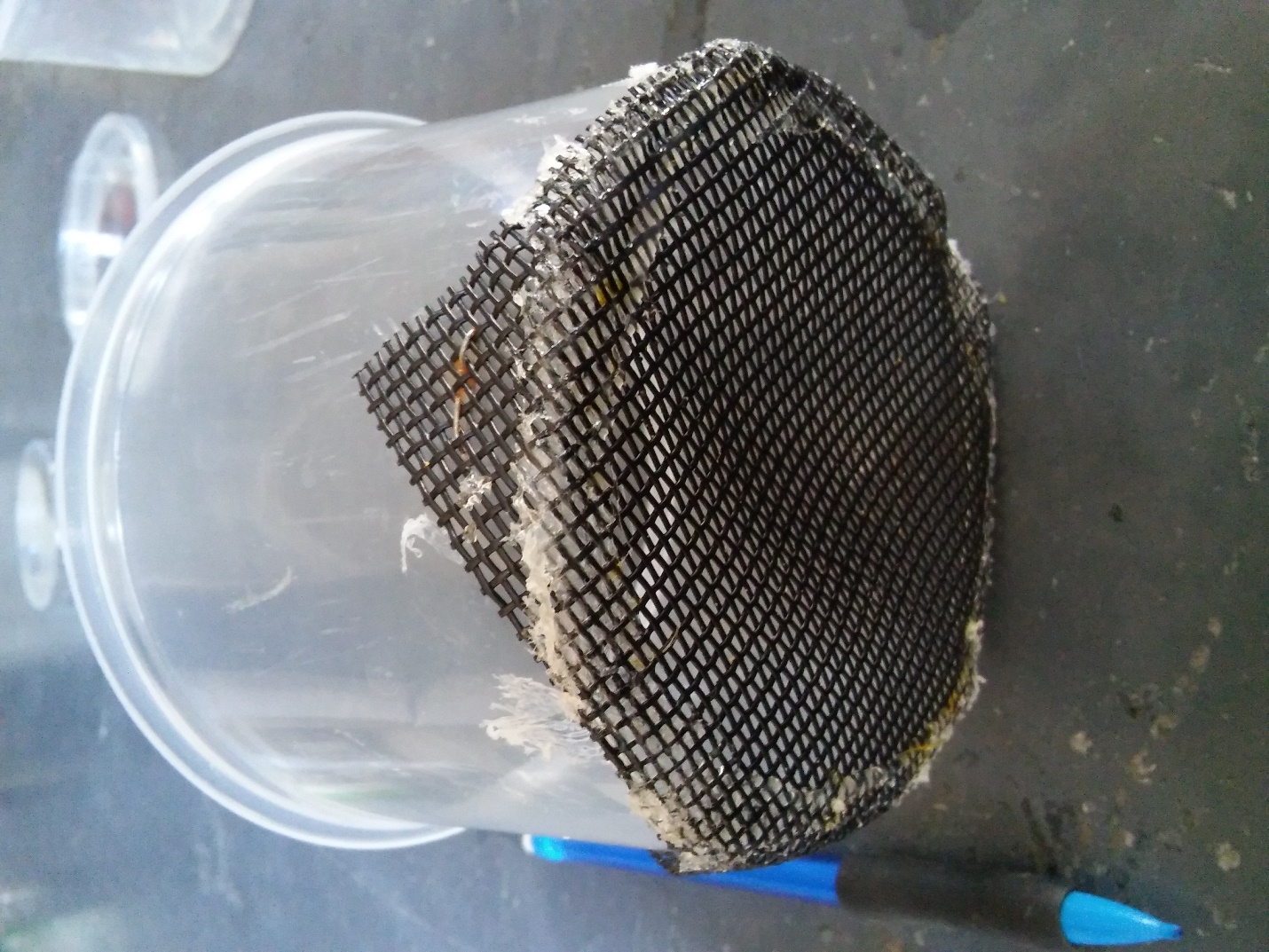
**

1. The inner cup was nested within an outer cup, into which was placed a petri dish with sucrose solution. A dental cotton wick was inserted into the solution through a hole in the top of the petri dish. The wire mesh bottom of the inner cup was placed against the top of the wick to allow bees access to the solution.

**
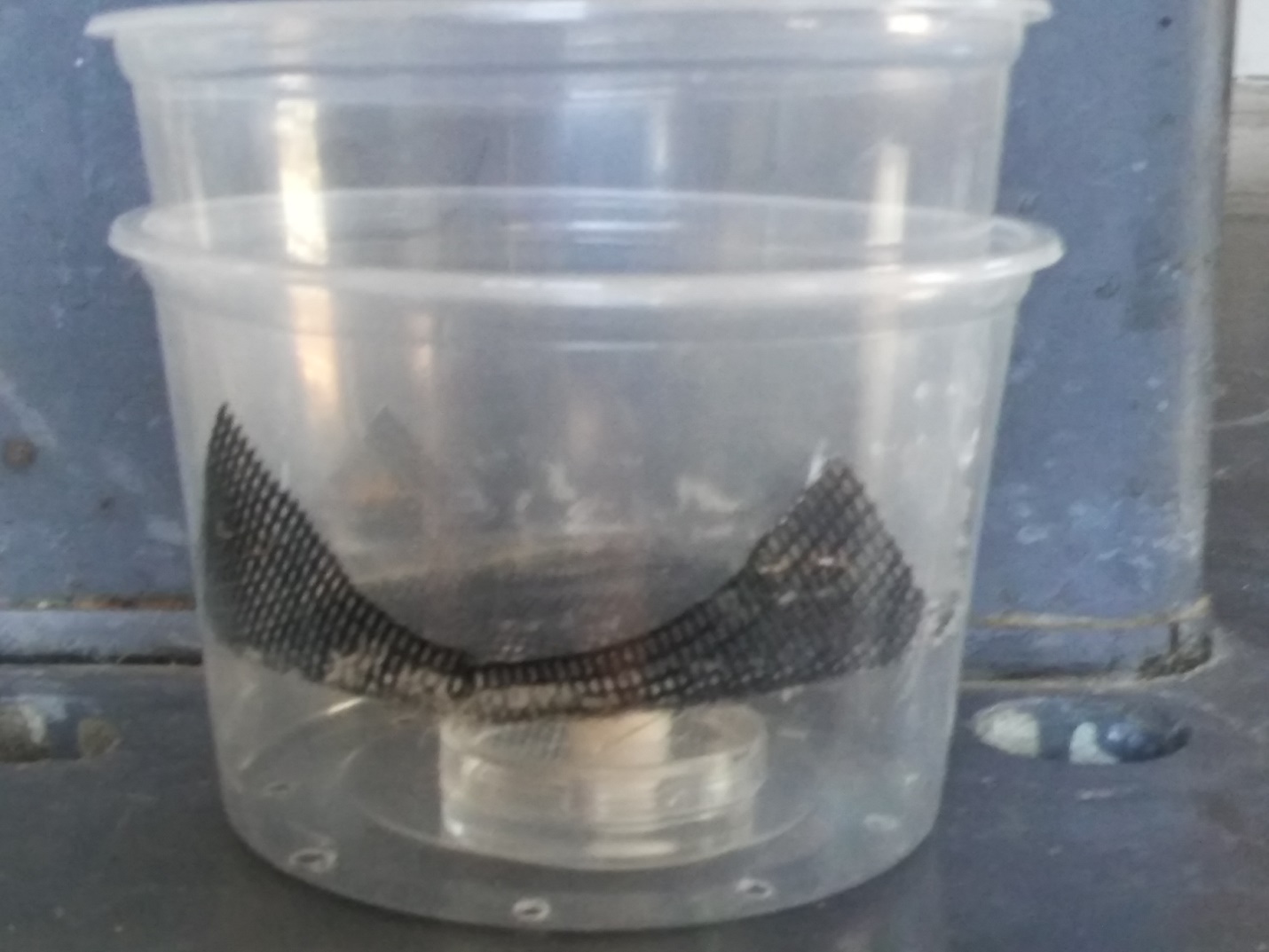
**

1. Birds-eye view of the Microcolony assembly

**
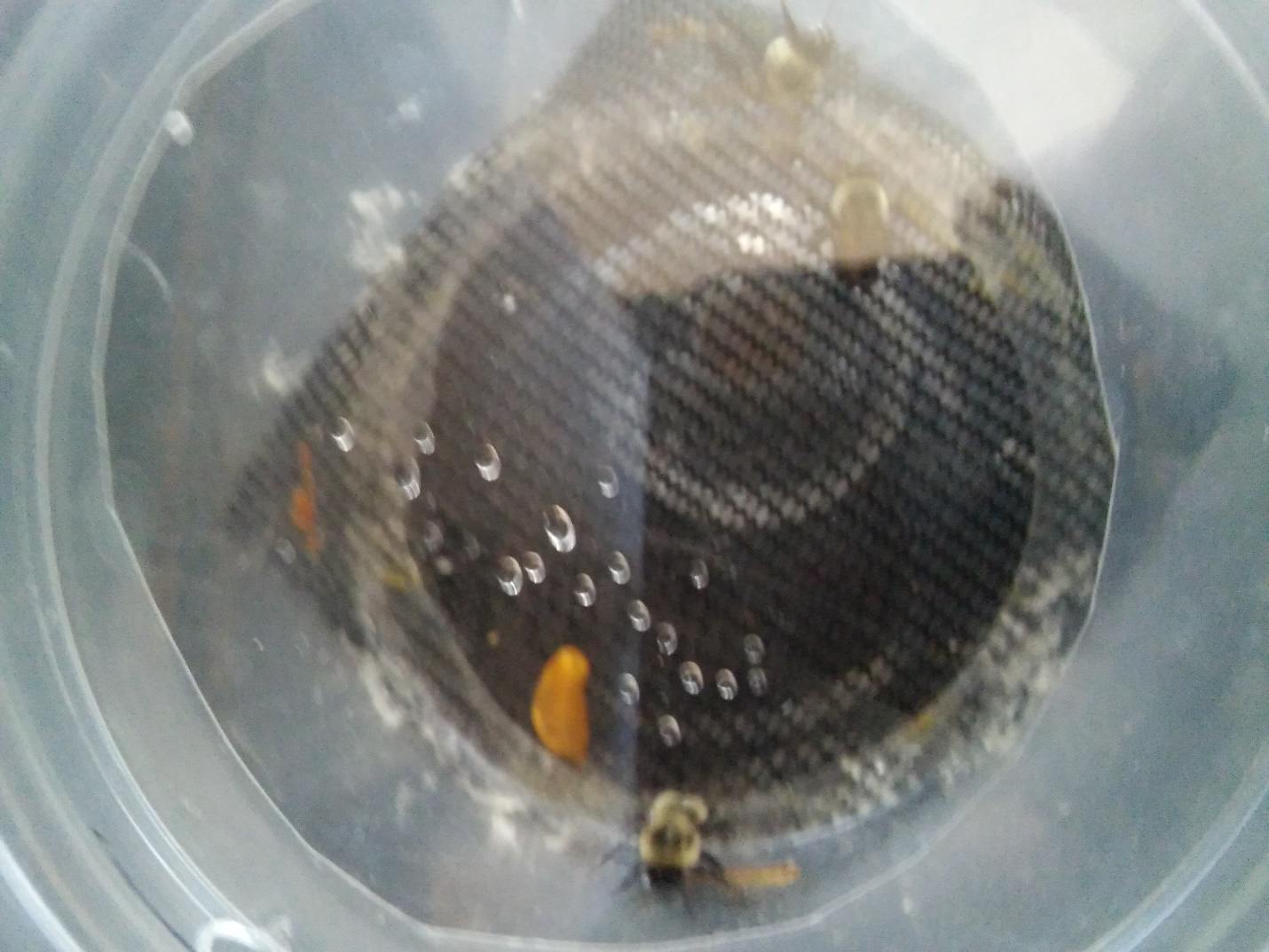
**

**Figure D.** Effects of anabasine on microcolony consumption of nectar and pollen in the Life History experiment.

Trendlines and shaded bands show model means +/- 1 standard error. Points show mean consumption at each time point.

1. Consumption of sucrose solution.
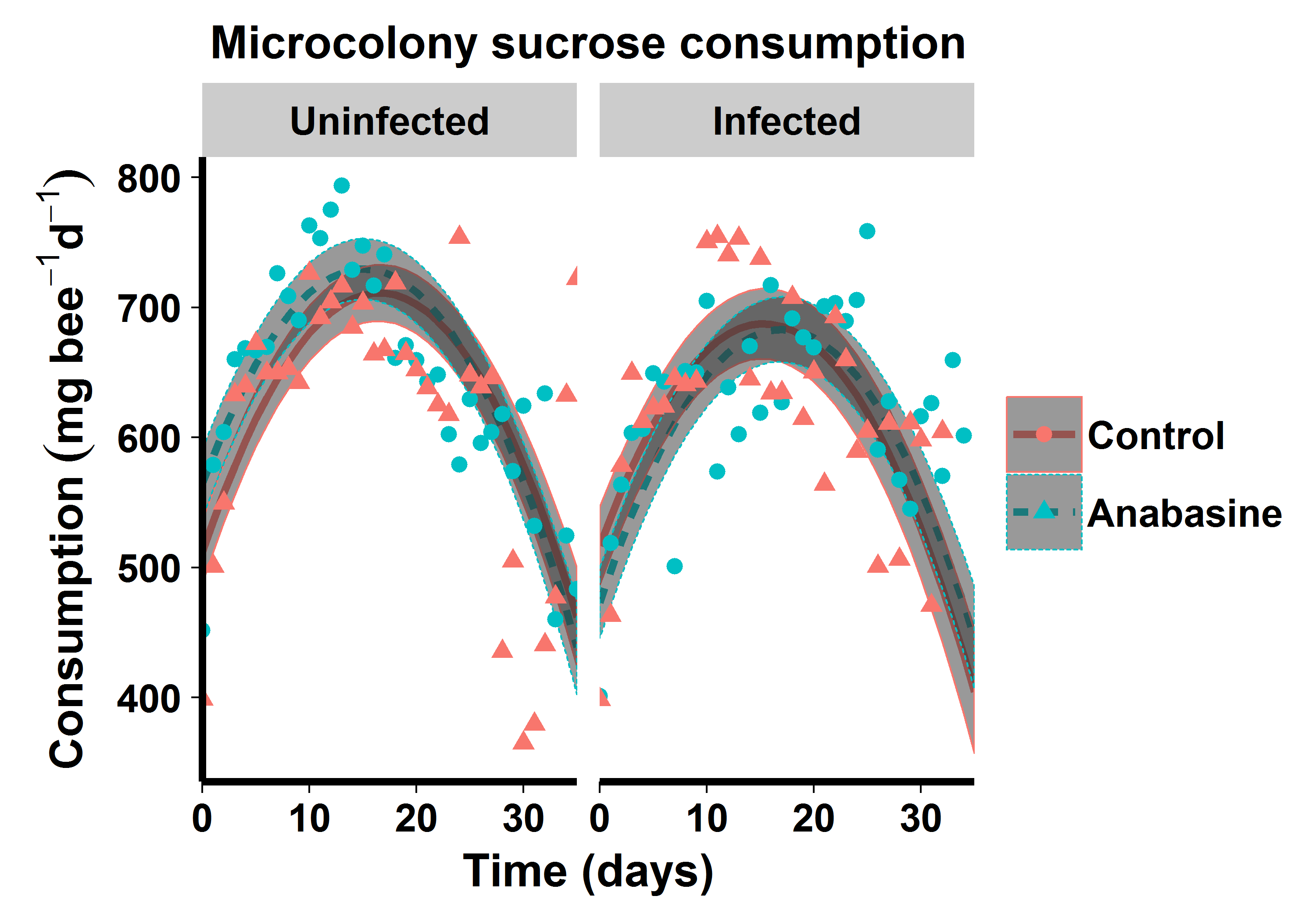


### Consumption of pollen.

Anabasine did not significantly affect pollen consumption, so results were averaged across ­anabasine treatments.
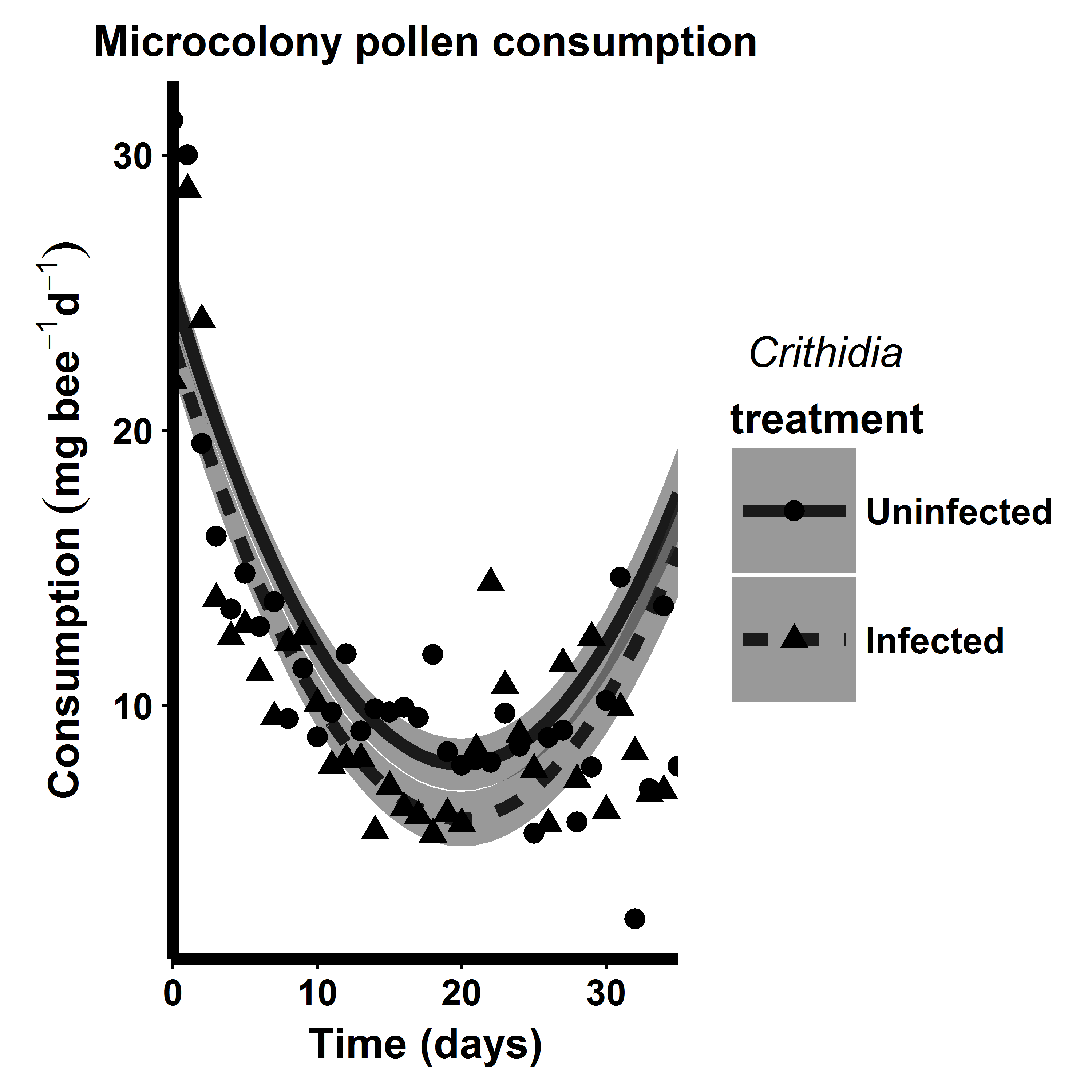


**Figure E.** Survival curves for individual bees fed anabasine (5 ppm) and control treatment solutions.

1. Parasite variation experiment. Sucrose control: red lines. Anabasine: blue lines. There were 46 deaths before dissection (at 7 d post-infection) among 602 total bees. X-axis shows number of days post-infection.


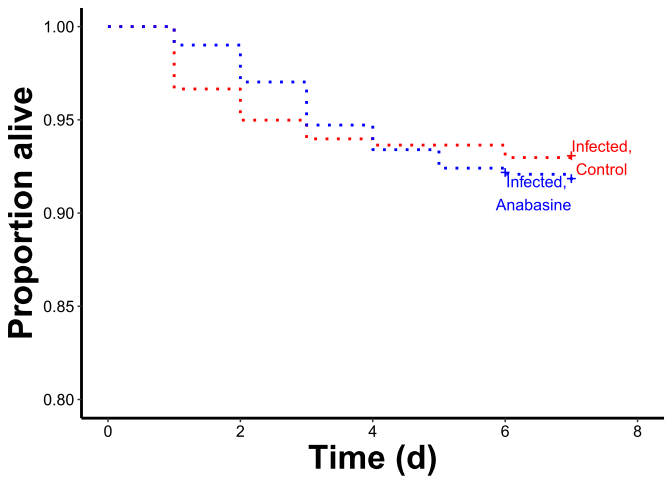


1. Pollen deprivation Experiment. Uninfected bees: solid lines. Infected bees: dotted lines. Sucrose control: red lines. Anabasine: blue lines. There were 34 deaths among 182 bees. X-axis shows number of days post-infection.


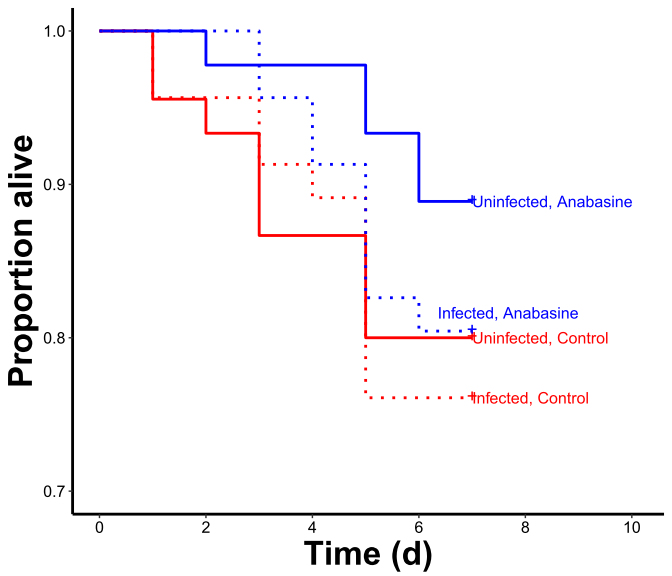


# Supplementary data files (key)

**S1 Data.** Microsoft Excel worksheet with separate sheets for Parasite variation (“Multi-lineage”, Life history (“Microcolony”), Host preference (“Preference”), and Pollen deprivation (No Pollen) experiments.

Explanations for column headers are as follows:

Bee.ID: Unique identifier for each bee in experiment

Colony.ID: Natal colony of experimental bee

Treatment: Anabasine treatment consisted of 5 ppm anabasine in 30% sucrose ("Anabasine"), or 30% sucrose alone ("Control")

Lineage: Parasite lineage used for inoculation

Mass: Mass in g at time of emergence from puparium

Dead.before.dissection: Whether bees died (1) or not (0) before dissection

Time.to.death : Number of days from infection to death or censoring. Recorded as "7" for bees that were dissected after 7 d.

Crithidia.count: Number of parasite cells in 0.02 µL gut extract
